# Supplementary material for: CARS Imaging Advances Early Diagnosis of Cardiac Manifestation of Fabry Disease
Source: Int J Mol Sci. 2022 May 11;23(10):5345. doi: 10.3390/ijms23105345 (PMC9142043; doi:10.3390/ijms23105345)
Supplement: Supplementary file 1 [file ijms-23-05345-s001.zip › ijms-1702146-supplementary.pdf]

International Journal of *Molecular Sciences*

Supplementary information

CARS Imaging Advances Early Diagnosis of Cardiac Manifestation of Fabry Disease

Elen Tolstik [1,*], Nairveen Ali [2], Shuxia Guo [2], Paul Ebersbach [1], Dorothe Möllmann [3], Paula Arias-Loza [4], Johann Dierks [1], Irina Schuler [1], Erik Freier [1], Jörg Debus [5], Hideo A. Baba [3], Peter Nordbeck [6], Thomas Bocklitz [2] and Kristina Lorenz [1,7,8,*]

1 Leibniz-Institut für Analytische Wissenschaften-ISAS-e.V., Bunsen-Kirchhoff-Str. 11, 44139 Dortmund, Germany; p.ebersbach@exeter.ac.uk (P.E.); johann.dierks@isas.de (J.D.); irina.schuler@isas.de (I.S.); erik.freier@uni-wuppertal.de (E.F.)
2 Leibniz Institute of Photonic Technology, Member of Leibniz Health Technologies, Albert-Einstein-Str. 9, 07745 Jena, Germany; nairveen@hotmail.com (N.A.); shuxia_guo@seu.edu.cn (S.G.); thomas.bocklitz@leibniz-ipht.de (T.B.)
3 Institute of Pathology, University Hospital Essen, Hufelandstr. 55, 45147 Essen, Germany; dorothe.moellmann@uk-essen.de (D.M.); hideo.baba@uk-essen.de (H.A.B.)
4 Department of Nuclear Medicine, Oberdürrbacher Str. 6, 97080 Wuerzburg, Germany; arias_p@ukw.de
5 Department of Physics, TU Dortmund University, Otto-Hahn-Str. 4a, 44227 Dortmund, Germany; joerg.debus@tu-dortmund.de
6 Department of Internal Medicine I, University of Würzburg, Oberdürrbacher Str. 6, 97080 Wuerzburg, Germany; nordbeck_p@ukw.de
7 Institute of Pharmacology and Toxicology, University of Würzburg, Versbacher Str. 9, 97078 Wuerzburg, Germany
8 Comprehensive Heart Failure Center, University Hospital of Würzburg, Am Schwarzenberg 15, 97078 Wuerzburg, Germany
* Correspondence: elen.tolstik@isas.de (E.T.); lorenz@toxi.uni-wuerzburg.de (K.L.)

Citation: Tolstik, E.; Ali, N.; Guo, S.; Ebersbach, P.; Möllmann, D.; Arias-Loza, P.; Dierks, J.; Schuler, I.; Freier, E.; Debus, J.; et al. CARS Imaging Advances Early Diagnosis of Cardiac Manifestation of Fabry Disease. *Int. J. Mol. Sci.* **2022**, *23*, 5345. https://doi.org/10.3390/ijms23105345

Academic Editors: Theophile Theophanides, Jane Anastassopoulou and Andreas Mavrogenis

Received: 12 April 2022
Accepted: 8 May 2022
Published: 11 May 2022

Publisher's Note: MDPI stays neutral with regard to jurisdictional claims in published maps and institutional affiliations.

Copyright: © 2022 by the authors. Licensee MDPI, Basel, Switzerland. This article is an open access article distributed under the terms and conditions of the Creative Commons Attribution (CC BY) license (https://creativecommons.org/licenses/by/4.0/).

Supplementary information

Reference Spectra Measurements of Gb3 and Lyso-Gb3 using Raman spectroscopy.

Aiming to prove our data analysis with the biomarker accumulations in FD cardiac tissues, we measured additionally the reference spectra of Gb3 and lyso-Gb3 substances in synthetic (crystalline) form. Due to lower spectral resolution no significant differentiation between both biomarkers could be obtained applying CARS microscopy and therewith no bimolecular changes could be found. To address this issue, we measured Gb3 and lyso-Gb3 at the confocal Raman micro-spectroscopy (see Material and Methods for the detailed description), which possesses higher spectral and lateral resolutions. Indeed, the Raman bands have slight shifts at CH stretching vibrations (e.g. at 2890 cm^{-1} and at 2930 cm^{-1}) providing detailed information. This advantage could support therapeutic strategies for FD and allows further investigation of molecular interactions (see "Discussion" section for more details on all advantages and disadvantages of Raman spectroscopy compared to CARS microscopy).

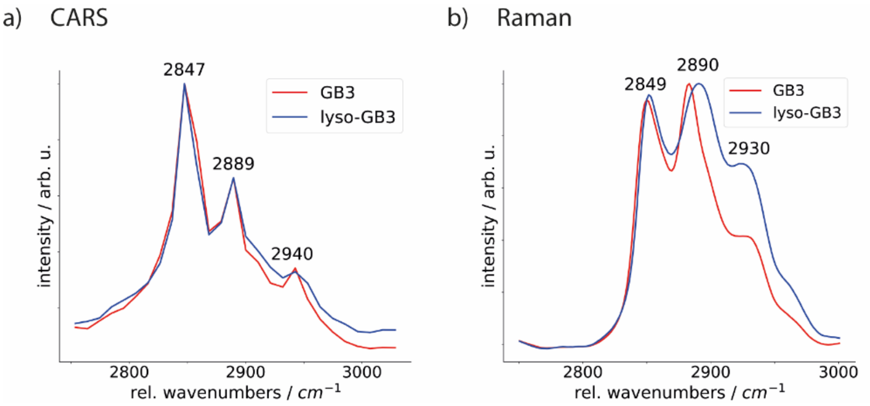

Figure S1. Reference spectra of globotriaosylceramide (Gb3, depicted in red) and its metabolite globotriaosylsphingosine (lyso-Gb3, depicted in blue) recorded using (**a**) CARS microscopy and (**b**) Raman micro-spectroscopy in C-H stretching region.
